# Non-specific vaginal discharge in reproductive -Age women: Response to Unani therapy

**DOI:** 10.6026/973206300222684

**Published:** 2026-04-30

**Authors:** Arjumand Shah, Arif Habibs, Huma Rafiq, Arsheed Iqbal, Nighat Ara

**Affiliations:** 1 Department of Medicine, Regional Research Institute of Unani Medicine, Naseembagh Campus-University of Kashmir, Srinagar, (Jammu and Kashmir) India

**Keywords:** Abnormal vaginal discharge, non-specific leucorrhoea, unani medicine, humoral imbalance, reproductive-age women, integrative gynaecology

## Abstract

Non-specific vaginal discharge in reproductive-age women represents a common clinical problem with limited effective therapeutic options, often persisting despite exclusion of identifiable infective or structural causes. The underlying pathophysiological basis remains poorly defined. Therefore, it is of interest to test the hypothesis that symptom persistence reflects a functional-inflammatory phenotype responsive to systemic humoral modulation. Hence, 80 women aged 18-45 years were evaluated longitudinally after standardized diagnostic exclusion using ordinal symptom scales. Treatment resulted in a significant reduction in discharge severity and associated symptoms, accompanied by decreased inflammatory markers and stable safety parameters (p < 0.001), supporting an idiopathic-inflammatory model amenable to targeted Unani therapy.

## Background:

Abnormal vaginal discharge is one of the most common gynaecological complaints among women of reproductive age and is associated with physical discomfort, psychological distress, impaired sexual health and reduced quality of life [1]. Vaginal discharge may be physiological; however, persistence, excessive quantity, offensive odour, or association with symptoms such as vulval pruritus, pelvic pain, backache and generalized weakness indicates a pathological condition requiring clinical evaluation [2]. Poor genital hygiene practices and delayed care-seeking behaviour further exacerbate morbidity, particularly in low-resource settings where reproductive health awareness is limited [3]. From a clinical standpoint, abnormal vaginal discharge (leucorrhoea) is classified into infective and non-infective causes [4]. While infective aetiologies such as bacterial vaginosis, vulvovaginal candidiasis and trichomoniasis are well characterized, a substantial proportion of women present with non-specific or non-infective leucorrhoea in which no identifiable pathogen are isolated [5]. This non-specific form has been linked to hormonal imbalance, nutritional deficiencies, chronic stress, pelvic congestion and inadequate genital hygiene, complicating diagnosis and long- term management [6]. Untreated or recurrent abnormal vaginal discharge has been associated with pelvic inflammatory disease, infertility, adverse pregnancy outcomes and increased susceptibility to sexually transmitted infections [7]. In the Unani system of medicine, excessive abnormal vaginal discharge is described as leukorrhea, a clinical entity documented since antiquity by physicians such as Hippocrates, Galen, Ibn Sina and Ismail Jurjani [8]. Leukorrhea is attributed to altered uterine temperament *(deranged temperament of the uterus)*, weakness of uterine retentive power *(reduced retaining capacity of the uterus)*, and predominance of morbid humours *(dominance of abnormal bodily humours)*, particularly phlegmatic and bilious states [9]. Classical descriptions of excessive discharge, pelvic heaviness, pruritus vulvae, fatigue and general debility closely parallel contemporary clinical presentations of non-specific leucorrhoea [10]. Unani therapeutics emphasizes restoration of physiological balance through correction of humoral derangement, strengthening of uterine function and improvement of general health using pharmacotherapy, dietary regulation and regimenal measures [11]. A classical polyherbal formulation listed in the National Formulary of Unani Medicine *Majoon Sohag Sonth*, has been traditionally prescribed for gynaecological disorders including Leukorrhea [12]. Experimental and pharmacological studies indicate that its constituent herbs possess uterine tonic, astringent, anti-inflammatory, antioxidant and nervine tonic properties that may contribute to reduction of excessive discharge and improvement of associated systemic symptoms [13]. Despite its traditional use, systematic clinical evaluation of *Majoon Sohag Sonth* using contemporary research methodologies remains limited, particularly for non-specific leucorrhoea [14]. Therefore, it is of interest to test the hypothesis that symptom persistence reflects a functional-inflammatory phenotype responsive to systemic humoral modulation

## Materials and Methods:

## Study design and reporting standards:

This prospective interventional clinical study was conducted and reported in accordance with the STROBE guidelines for observational components and CONSORT principles for reporting interventional outcomes, particularly with respect to participant flow, outcome assessment and statistical analysis of repeated measures.

## Participants:

Women aged 18-45 years presenting with complaints of excessive vaginal discharge were screened for eligibility. Women aged 18-45 years who were sexually active and presented to the outpatient department with complaints of abnormal vaginal discharge were eligible for inclusion. Participants were required to report vaginal discharge with or without associated symptoms, including vulval itching, low backache, generalized weakness, burning micturition, or abnormal vaginal odour. Women who were pregnant, lactating, or postmenopausal were excluded. Additional exclusion criteria included the presence of systemic comorbidities such as hypertension or diabetes mellitus; known structural or pathological gynaecological conditions (including uterine fibroids, endometrial polyps, ovarian cysts or tumours, uterovaginal prolapse, or malignancy); abnormal findings on pelvic ultrasonography; and current use of oral contraceptive pills or intrauterine contraceptive devices. Participants with laboratory-confirmed sexually transmitted infections, including human immunodeficiency virus (HIV) infection or syphilis (VDRL positivity), those receiving long-term pharmacotherapy and women diagnosed with acute, acute-on-chronic, or chronic pelvic inflammatory disease according to the Centers for Disease Control and Prevention (CDC) criteria were also excluded.

## Intervention study drug:

## *Majoon Sohag Sonth* (semi-solid Unani/herbal formulation):

The treatment drug documented in the National Formulary of Unani Medicine, Part II, 2008 was a classical Unani Pharmocopoeial formulation *Majoon Sohag Sonth*. The formulation is a semisolid preparation *(majoon)* combines herbs traditionally used to restore humoral balance, improve uterine tone and support reproductive health. *Zingiber officinale* (ginger) exhibits anti-inflammatory properties [17, 19] and has been shown to reduce uterine-associated pain and inflammation in clinical settings, suggesting potential benefit in pelvic discomfort and inflammatory imbalance. *Withania somnifera* (ashwagandha) [15] has demonstrated effects on reproductive system function and reduction of oxidative stress, supporting its role in reproductive and systemic tonic action. *Asparagus racemosus* (shatavari) is recognized for its reproductive health benefits, including potential hormonal modulation and enhanced female reproductive function [16]. Astringent and tissue-toning components such as *Acacia arabica* gum also show potential in gynaecological applications [18].These collective actions align with the Unani therapeutic goals of strengthening uterine retentive power and correcting morbid humours in *Leukorrhea.*

## Dosage and administration:

Participants received 7 grams of *Majoon Sohag Sonth* orally twice daily for duration of four (4) weeks. No concomitant medications were permitted during the study period to avoid confounding effects on outcome assessment.

## Outcome measures:

For the purpose of statistical evaluation, symptoms were categorised according to predefined options for each variable. The primary outcomes were changes in clinical symptoms related to excessive vaginal discharge, including quantity, type, odour, vulval itching, backache and generalized weakness. These outcomes were recorded at baseline (T1) and after completion of the intervention period (T2) using standardized categorical scales to ensure consistency across time points. Participants were evaluated at three time points. At baseline (Day 0), a complete clinical and laboratory assessment was conducted. The first follow-up at Week 2 included clinical assessment, symptom scoring, vital signs measurement, Nabz examination and evaluation of medication compliance. The second follow-up at Week 4 involved a repeat of the complete clinical and laboratory assessment identical to baseline, along with final symptom scoring and a global assessment of treatment response. A window of ± 3 days was allowed for rescheduling follow-up visits in case of missed appointments. Medication compliance was closely monitored throughout the study. This included counting returned boxes of semi-solid electuary at each visit, documenting the quantity dispensed and returned, recording patient-reported missed doses along with reasons and grading overall compliance as excellent, good, or poor. Secondary outcomes included changes in haematological and biochemical parameters to assess systemic safety of the intervention.

## Laboratory investigations:

Haematological parameters (haemoglobin, total leukocyte count, erythrocyte sedimentation rate) and biochemical parameters (liver and renal function tests) were measured at baseline and after intervention using standard laboratory procedures.

## Sample size justification:

As this was an exploratory clinical study aimed at evaluating changes in multiple categorical clinical outcomes over time, a formal a priori sample size calculation was not performed. A pragmatic sample size of 80 participants was considered adequate based on feasibility and prior similar clinical studies assessing symptom-based outcomes in gynaecological conditions. This sample size provided sufficient observations to detect clinically meaningful within-subject changes over time using repeated-measures analytical methods such as generalized estimating equations.

## Statistical analysis:

Descriptive statistics were used to summarize baseline characteristics. Continuous variables are presented as mean ± standard deviation, while categorical variables are expressed as frequencies and percentages. Categorical clinical outcomes assessed at baseline and follow-up were analysed using generalized estimating equations (GEE) with a multinomial probability distribution and cumulative logit link function, appropriate for ordinal outcome data. Time was treated as a within-subject factor and clustering of repeated measurements within individuals was accounted for using an exchangeable working correlation structure. The statistical significance of model effects was assessed using Wald χ^2^ statistics and results are presented as odds ratios with 95% confidence intervals. Comparisons of haematological and biochemical parameters before and after intervention were performed using appropriate paired statistical tests based on data distribution. A two-sided p-value <0.05 was considered statistically significant. All analyses were performed using standard statistical software.

## Confidentiality:

All participant information was maintained confidentially. Only the research team and authorized personnel (ethics committee members, institutional or regulatory authorities) had access to participant data. Participants were assigned unique identification numbers and personal identifiers were protected in accordance with applicable data protection regulations.

## Quality assurance:

Standard operating procedures (SOPs) were followed for all study-related activities including participant screening, consent procedures, drug dispensing, clinical assessments, sample collection, laboratory testing, data recording and adverse event reporting. Regular monitoring visits were conducted to ensure protocol adherence and data integrity.

## Results and Discussion:

Odds ratios were derived from generalized estimating equation (GEE) models with multinomial distribution and cumulative logit link, adjusted for within-subject correlation using an exchangeable working correlation structure. Start (T1) served as the reference category. Eighty women presenting with excessive vaginal discharge were included in the final analysis. The mean age of participants was 29.46 ± 8.37 years, with mean symptom duration of 9.73 ± 8.39 months at baseline, indicating a predominantly young reproductive-age population with chronic or recurrent complaints. At enrolment, most participants reported moderate to profuse vaginal discharge (80.0%), yellow-coloured discharge (71.3%) and offensive odour (72.5%). On gynaecological examination, a bulky uterus was noted in 63.8% of cases, while inflammatory or vaginosis-related changes of the vaginal wall were observed in 72.5% (Table 1 - see PDF). These findings reflect a substantial symptom burden and are consistent with epidemiological and hospital-based reports identifying abnormal vaginal discharge as one of the most common gynaecological complaints among reproductive-age women [1, 3]. Beyond physical discomfort, such symptom clusters are known to significantly impair quality of life, daily functioning and psychosocial well-being, particularly when symptoms are persistent or recurrent [1,14]. Changes in clinical parameters from baseline (T1) to follow-up (T2) were analysed using generalized estimating equation models to account for repeated measurements. A highly significant improvement was observed in the primary outcome-quantity of vaginal discharge-at follow-up (Wald χ^2^ = 188.89, p < 0.001), with a marked reduction in the likelihood of severe discharge (OR = 51.52; 95% CI: 29.37-90.38). The type of discharge also showed substantial improvement, with significantly lower odds of abnormal discharge patterns at follow-up (OR = 15.21; 95% CI: 8.29-27.88; p < 0.001). Offensive odour demonstrated a statistically significant reduction over time (OR = 8.77; 95% CI: 1.37-56.26; p = 0.022). In addition, associated symptoms such as vulval itching, backache and generalized weakness showed consistent and clinically meaningful improvement, with statistically significant reductions across all parameters (p < 0.001 for each; Table 2 - see PDF). Collectively, these findings indicate a robust and sustained therapeutic response following the intervention. From a contemporary biomedical perspective, abnormal vaginal discharge is often attributed to microbial dysbiosis, including bacterial vaginosis, vulvovaginal candidiasis and Trichomonas vaginalis infection [5, 7 and 20]. However, a considerable subset of women present with non-specific or non-infective discharge, in which microbiological investigations fail to identify a causative pathogen. In such cases, contributory factors include hormonal fluctuations and uterine functional disorders [4], nutritional deficiencies and general debility [1].

Psychosocial stress and autonomic dysregulation [16], contraceptive use and reproductive behavioural factors [3] and broader diagnostic challenges inherent to non-specific vaginitis [2^22^]. This clinical heterogeneity complicates standardized management and frequently results in repeated empirical antimicrobial use, often with limited long-term benefit [14, 21 and 23]. The marked improvement observed in discharge quantity, type, odour and associated systemic symptoms following administration of *Majoon Sohag Sonth* suggests that the formulation exerts effects beyond simple symptomatic relief. The magnitude and consistency of response support a role in modulating underlying non-specific pathophysiological mechanisms rather than targeting infective causes alone. This is particularly relevant in women with non-specific genital discharge, where inflammatory and regulatory dysfunction predominates over overt infection [13, 14 and 22]. Within the Unani medical framework, non-specific vaginal discharge corresponds to Leukorrhea is attributed to a deranged (or abnormal) temperament of the uterus, weakness of the uterine retentive faculty, and accumulation of morbid humours [8, 9]. Classical Unani literature describes clinical features such as excessive vaginal discharge, pelvic heaviness, pruritus vulvae, fatigue and general debility-features those closely parallel contemporary descriptions of non-specific vaginal discharge [10, 11]. The high prevalence of bulky uterus and vaginal wall inflammatory changes in the present cohort further supports a functional or non-infective aetiology, aligning with modern gynaecological descriptions of non-specific genital discharge [4, 14 and 22]. Haematological and biochemical parameters remained largely stable throughout the study period, with significant reductions observed only in inflammatory markers and select liver enzymes (Table 3 - see PDF). The observed reduction in erythrocyte sedimentation rate, alongside stability of hepatic and renal function indices, indicates a favourable systemic safety profile and suggests attenuation of low-grade inflammation. These findings are pharmacologically plausible, given existing experimental and clinical evidence supporting the anti- inflammatory, uterine tonic and adaptogenic properties of key constituents of *Majoon Sohag Sonth*. Notably, *Zingiber officinale* has demonstrated immunomodulatory and anti-inflammatory effects [17, 19],while *Withania somnifera* is known for its regulatory and adaptogenic influence on reproductive and systemic function [15, 16]. Such systemic actions are particularly pertinent in non-specific vaginal discharge, where inflammatory dysregulation and functional imbalance rather than infection dominate the clinical picture [14, 22]. Taken together, the present findings support a data-driven reinterpretation of non-specific vaginal discharge as a predominantly non-infective, functional-inflammatory condition that may be more effectively managed through systemic therapeutic modulation rather than routine antimicrobial therapy [21, 22]. The integration of validated Unani formulations into contemporary gynaecological practice may therefore offer a rational, safe and culturally acceptable management strategy, especially in low-resource settings where recurrent symptoms, antimicrobial overuse and limited access to specialized care remain persistent challenges.

## Conclusion:

*Majoon Sohag Sonth* produced significant clinical improvement in the severity and characteristics of vaginal discharge, along with associated systemic symptoms, while showing an acceptable safety profile. These findings support its role as a rational and effective therapeutic option for the management of non-specific vaginal discharge in reproductive-age women.
